# Metformin Repurposing for Parkinson Disease Therapy: Opportunities and Challenges

**DOI:** 10.3390/ijms23010398

**Published:** 2021-12-30

**Authors:** Francesco Agostini, Anna Masato, Luigi Bubacco, Marco Bisaglia

**Affiliations:** 1 Department of Biology, University of Padova, 35121 Padova, Italy; francesco.agostini.6@phd.unipd.it (F.A.); anna.masato@unipd.it (A.M.); 2 Center Study for Neurodegeneration (CESNE), University of Padova, 35121 Padova, Italy

**Keywords:** Parkinson disease, metformin, neuroprotection, AMPK, epidemiology, bioavailability

## Abstract

Parkinson disease (PD) is a severe neurodegenerative disorder that affects around 2% of the population over 65 years old. It is characterized by the progressive loss of nigrostriatal dopaminergic neurons, resulting in motor disabilities of the patients. At present, only symptomatic cures are available, without suppressing disease progression. In this frame, the anti-diabetic drug metformin has been investigated as a potential disease modifier for PD, being a low-cost and generally well-tolerated medication, which has been successfully used for decades in the treatment of type 2 diabetes mellitus. Despite the precise mechanisms of action of metformin being not fully elucidated, the drug has been known to influence many cellular pathways that are associated with PD pathology. In this review, we present the evidence in the literature supporting the neuroprotective role of metformin, i.e., autophagy upregulation, degradation of pathological α-synuclein species, and regulation of mitochondrial functions. The epidemiological studies conducted in diabetic patients under metformin therapy aimed at evaluating the correlation between long-term metformin consumption and the risk of developing PD are also discussed. Finally, we provide an interpretation for the controversial results obtained both in experimental models and in clinical studies, thus providing a possible rationale for future investigations for the repositioning of metformin for PD therapy.

## 1. Introduction

Parkinson disease (PD) is the second most common and fastest-growing neurological disorder in the world [1]. It affects more than 6 million people worldwide and it is estimated to increase to more than 12 million by 2040 [1]. Motor symptoms, which represent the major clinical feature of PD, are associated with the preferential loss of dopaminergic neurons in the substantia nigra pars compacta (SNpc). Another pathological hallmark of the disease is the presence in the surviving neurons of intracellular proteinaceous inclusions, referred to as Lewy bodies, mainly composed of α-synuclein (α-syn). Mutations in the *SNCA* gene, coding for the protein α-syn, have been associated with familial forms of PD [2], highlighting the role of the protein in the pathological mechanisms underlying the disease.

PD is still an incurable disorder and the currently available therapeutic approaches focus on stimulation of dopaminergic signaling, such as levodopa (L-DOPA), DOPA decarboxylase inhibitors, catechol-O-methyltransferase inhibitors, dopamine agonists, and inhibitors of the enzyme monoamine oxidase type B [3]. Unfortunately, all these treatments can only provide symptomatic relief but they do not hamper the clinical and pathological progression of the disorder. As a consequence, the development of new disease-modifying therapies is one of the major challenges for the treatment of PD. To design disease-modifying therapies is usually long, expensive, and highly risky, considering that it takes an average of 13–15 years with an estimated cost of bringing a new molecule to market of around 2.6 billion USD. Moreover, only ~10% of drugs entering clinical trials make it to the market [3,4]. In light of all these drawbacks, an alternative strategy is drug repurposing, also known as drug repositioning, which is the evaluation of existing drugs for new therapeutic purposes, outside their original clinical indication [5]. The advantage of this strategy is the possibility to bypass several preclinical and clinical phases, by the use of molecules whose pharmacokinetics, safety, and toxicology profiles have been already established.

Therefore, in the quest for new potential therapeutic compounds against PD progression, the knowledge of the pathological mechanisms involved in its etiopathogenesis and the comparative analysis for their potential roles in other disorders could help speed up drugs’ repurposing. In this frame, we recently analyzed the literature data supporting a direct association between type 2 diabetes mellitus (T2DM) and an increased probability to develop PD, suggesting a potential molecular mechanism underlying such a correlation [6]. On the basis of this analysis, among the different marketed anti-diabetic drugs, metformin was proposed as a promising candidate for novel PD therapy. As recently reviewed, metformin is used as the first-line treatment of T2DM worldwide, being approved by the FDA in 1995 [7]. It is a low-cost medication, which is generally well tolerated with minimal side effects. Over time, the benefits of metformin have been described beyond diabetes and its potential application to treat various diseases has been proposed [8], making metformin one of the most promising drugs for repurposing.

In this review, we will analyze the therapeutic potentials of metformin repurposing for PD, considering the main mechanisms of action that would provide a neuroprotective effect. At the same time, we will present a critical appraisal of the use of metformin in both experimental PD models and clinical studies, highlighting the challenge in data interpretation and evaluation of metformin effectiveness as a disease modifier for PD.

## 2. Potential Neuroprotective Mechanisms of Action of Metformin

The antidiabetic action of dimethylbiguanide (metformin) is principally related to the inhibition of hepatic gluconeogenesis, the promotion of peripheral glucose uptake, the increased insulin sensitivity, and its anti-glycating action, which altogether stabilize patients’ glycemia [9].

In recent years, metformin has gained increasing interest not only for its glucose-lowering capacity but also due to the possible beneficial effects in different pathological conditions, including neurodegenerative diseases. The impact of metformin on neuronal homeostasis has been increasingly studied in both in vitro and in vivo models (Table 1 and Table 2), observing a general improvement in the lifespan of the animal PD models, a rescue of dopaminergic neuron loss, and motor phenotypes. However, the precise effect of the drug is still under debate and needs further characterization.

**Figure 1:**
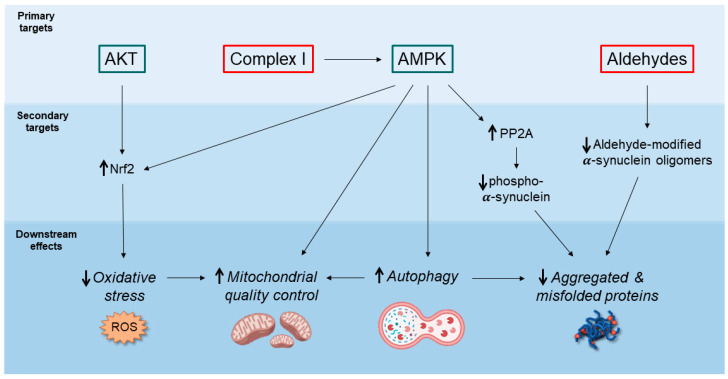
Potential neuroprotective mechanisms of action of metformin. This schematic diagram illustrates the most relevant molecular pathways and the cellular processes affected by metformin. In the upper section, the primary targets activated by metformin (AKT and AMPK) are highlighted in green. Metformin direct targets that are inhibited by the drug (Complex I and Aldehydes) are indicated in red. In the lower sections the secondary molecular targets are represented together with the downstream effects of metformin, which include the increase of autophagic activity, the reduction of aggregated or misfolded proteins, the decrease of ROS, and the improvement of mitochondrial functions. All these outcomes may account for the potential neuroprotective action of metformin.

Here, the most relevant cellular pathways that are modulated by metformin are summarized in Figure 1 and they will be discussed in the following paragraphs.

### 2.1. AMPK-Mediated Autophagy Activation

One of the most studied and relevant effects of metformin is the modulation of the AMP-activated protein kinase (AMPK) activity. AMPK is a ubiquitously expressed kinase protein that is considered a sensor of cellular metabolic status and plays a crucial role in maintaining cellular energetic homeostasis [27,28]. AMPK function is affected by different stimuli, like changes in the intracellular calcium level or imbalance in the oxygen reactive species (ROS) concentration [27,29]. Moreover, the major stimulus that is known to modulate AMPK activity is the variation of the ATP/AMP ratio, as the increase of AMP concentration results in its activation, due to the binding between AMPK and AMP. This interaction causes conformational changes in the protein that becomes more prone to be phosphorylated by the upstream activator kinases, in particular the Liver Kinase B1 (LKB1) [27]. In turn, AMPK can influence several important pathways, coordinating key cellular processes, such as autophagy, cell growth, and mitochondrial quality control [28]. Interestingly, some of the molecular mechanisms affected by AMPK function are crucial for neuronal cell survival and are known to be dysregulated in different neurodegenerative disorders, including PD. Thus, targeting AMPK to enhance its activity is considered a promising neuroprotective strategy [29,30]. Noteworthy, many studies demonstrated, both in vitro and in vivo, that metformin can activate AMPK [10] through a mechanism that is still under debate [31]. Importantly, this effect has been analyzed also in the neurodegeneration context, exploiting a cellular model of Alzheimer Disease (AD) characterized by the accumulation of amyloid-beta (Aβ) deposits. In this frame, the activation of AMPK mediated by metformin has been shown to increase cell viability and rescue mitochondrial defects by increasing mitochondrial mass and ameliorating mitochondrial function. These beneficial effects are associated with the activation of several AMPK downstream proteins including Bcl-1, CREB, and PGC1α [10].

Among the processes regulated by AMPK, the activation of autophagy is one of the best-studied. This mechanism has been analyzed in hippocampal rat neurons in which metformin was shown to activate AMPK, increasing neuroprotection through the mechanistic target of the rapamycin (mTORC1) pathway [32]. In this frame, the AMPK-mediated inhibition of mTORC1 has been described to be beneficial to maintain neuronal homeostasis by promoting autophagic activity and lysosomal biogenesis [18,33,34,35]. The AMPK-mTORC1 pathway has been proven to be activated by metformin also in vivo in *C. elegans* where the treatment with the drug increased lifespan through the induction of the lysosomal pathway [18].

*Drosophila melanogaster* has also been used as an in vivo model to evaluate the effect of metformin on the AMPK pathway. The oral administration of the drug was demonstrated to increase the levels of threonine 172 phosphorylation, which is necessary for the activation of the protein [20]. Although the use of metformin was not analyzed in fruit fly models for neurodegenerative disorders, increasing AMPK activity is beneficial in fly models of several diseases, suggesting the possibility to target this kinase with metformin as a therapeutic approach [36,37].

The metformin-mediated AMPK activation and the crucial role of AMPK activity have been widely investigated also in mammalian models, where it was shown that increasing AMPK activity may protect cells against different stress stimuli. For instance, in mice, metformin increases lifespan through the activation of AMPK [22].

Despite these positive observations, several questions about the activity of metformin are still unsolved and need further investigation in order to avoid possible negative effects. For example, the mechanism by which metformin modulates the activity of AMPK is still unclear. The hypothesis that metformin could directly activate AMPK was excluded long ago when the incubation of purified AMPK from rat liver with metformin failed to activate the kinase [12], indicating that the drug indirectly activates AMPK, likely affecting upstream regulators of the protein. Interestingly, metformin failed to activate AMPK in mice knockout for LKB1, an upstream activator of AMPK able to phosphorylate the protein at the T172 residue [38]. Therefore, it appears that the treatment can influence one or more upstream pathways.

### 2.2. Decreased Accumulation of α-Synuclein Pathological Species

Autophagy activation represents a good strategy to reduce the accumulation of toxic protein aggregates, which are detected at the histological level in different neurodegenerative diseases. In PD, the progressive accumulation of α-syn in neurons due to oxidative stress, certain post-translational modifications, or impairment of protein degradation systems generates α-syn neurotoxic oligomers that are known to affect several cellular pathways [39]. Hence, the upregulation of the autophagic pathway by metformin might counteract α-syn pathology by rapidly disposing of the α-syn aggregates, as recently shown in a *C. elegans* PD model exposed to 6-hydroxydopamine (6-OHDA), where the treatment with metformin resulted in a reduction of both α-syn aggregation and dopaminergic neuron loss [23].

It has been also demonstrated that metformin can reduce the levels of phosphorylated α-syn at serine 129 (α-syn pSer129), which is usually considered a read-out of pathological and aggregated α-syn species. Specifically, both in vitro and in vivo, metformin administration resulted in time- and dose-dependent activation of protein phosphatase 2A (PP2A), which is known to mediate α-syn dephosphorylation [15]. According to the authors, PP2A activation by metformin can happen via both AMPK-dependent and -independent pathways. On the same line, metformin administration to mice previously injected with 1-methyl-4-phenyl-1,2,3,6-tetrahydropyridine (MPTP) significantly reduced α-syn pSer129 levels through the activation of PP2A [24]. In this model, metformin-mediated decreased levels of pathological α-syn together with autophagy activation, upregulation of neurotrophic factors (BDNF, GDNF), and downstream signaling pathways (Akt, Erk1/2) resulted in restored dopamine concentration at the striatum and rescue of motor performance [24], thus underlying the multiple neuroprotective mechanisms of action of this drug.

On a different note, due to the ability of the guanidino group and the primary amine present in metformin structure to act as a scavenger of the aldehyde moiety of methylglyoxal (MGO), metformin has been used as an anti-glycating agent in T2DM therapy to prevent the accumulation of proteins modified by MGO and advanced glycating end products (AGEs) [40]. Interestingly, MGO has been demonstrated to covalently modify α-syn and trigger its oligomerization in several in vitro and in vivo models, exacerbating PD-like neurodegeneration [41]. In this frame, the administration of aminoguanidine, an analog of metformin, reduced α-syn aggregation and promoted its clearance via autophagy in MGO-treated cells and rescued the motor impairment observed in MGO-treated flies overexpressing the human α-syn [41]. Hence, we recently speculated that metformin could provide a similar scavenging activity towards MGO by preventing the accumulation of α-syn neurotoxic aggregates, but this could be extended to other aldehydes of neuropathological relevance in PD, i.e., aldehydes derived from oxidative stress and lipid peroxidation (4-hydroxynonenal (4-HNE), malondialdehyde (MDA)) or aldehydic molecules that accumulate from altered monoamine catabolic pathways (3,4-dihydroxyphenylacetaldehyde, 3,4-dihydroxyphenylglycoaldehyde, 5-hydroxyindole-3-acetaldehyde) [42]. Accordingly, a recent paper investigated the neuroprotective effect of metformin in a PD mouse model based on rotenone-induced dopaminergic neuron death [25]. The authors demonstrated that metformin co-administration with rotenone significantly reduced the nigral levels of 4-HNE and MDA, together with decreased α-syn accumulation and dopaminergic neuron degeneration in the SNpc [25]. Although the authors did not investigate it directly, the reduced α-syn buildup might derive, at least in part, from a scavenging activity of metformin towards lipid peroxidation products, preventing α-syn modification and oligomerization.

It is worth mentioning that the spreading of aggregated and phosphorylated α-syn toxic species has a crucial role in the progression of PD pathology [43,44,45]. Several mechanisms have been described to mediate the cell-to-cell transmission of pathological α-syn (trans-synaptic transmission, extra-cellular vesicles, and endocytosis) across diverse brain regions [46]. Moreover, according to the Braak hypothesis, the primary events of α-syn aggregation and spreading originate outside the brain, either in the olfactory bulb or the gastrointestinal tract, further propagating through the cranial nerves from the peripheral nervous system to the brainstem and the other vulnerable areas in the CNS (i.e., SNpc and the striatum) [47,48,49]. In this context, both the autophagic enhancing activity of metformin and the ability to degrade α-syn neurotoxic aggregates before propagation may be of great value to restrain α-syn spreading and to slow down the disease progression. Importantly, the accumulation of α-syn aggregates is a feature shared by different synucleinopathies, which include, besides PD, Dementia with Lewy Bodies (DLB), and Multiple System Atrophy (MSA) [50]. Therefore, it is plausible to hypothesize that the treatment with metformin may be beneficial not only in the context of PD but also in other pathologies characterized by α-syn aggregation. These pathologies have been suggested to display structurally different α-syn fibrillary patterns or strains as well as different cell types, both neuronal and glial, in which the aggregates are found. All these issues may account for the heterogeneity of the clinical phenotypes associated with α-syn accumulation [51]. In this frame, it would be crucial to characterize in detail the action of metformin in the diverse brain regions and cellular subpopulations to assess whether the neuroprotective effect of the drug varies among the specific cellular target or α-syn conformation, thus resulting in a differential outcome according to the diverse synucleinopathy.

### 2.3. Inhibition of Mitochondrial Complex I and Regulation of Mitochondrial Dynamics

Metformin has been widely studied as a molecule able to influence mitochondrial functions. In this frame, more than 20 years ago it was observed that in rats treated with metformin, the molecule slowly penetrated across the inner mitochondrial membrane and accumulated inside the organelles, where it appeared to cause the inhibition of complex I activity possibly through a direct interaction [11]. Even though this effect is one of the most studied and established, the precise mechanism of metformin’s function at the mitochondrial level is still under debate [52]. It is worth mentioning that while a mild inhibition of complex I may result in beneficial effects for cell homeostasis, the inhibition of the complex I may also be detrimental, in particular in the context of PD. In fact, the reduction of the mitochondrial respiratory chain function may determine a decrease in the ATP/ADP ratio, resulting in a relatively higher concentration of ADP that can bind AMPK and activate the protein [11]. However, in PD the decrease of complex I activity has been proposed to be one of the triggering factors for the disease [53]. Accordingly, some reports highlighted the possibility that the metformin-mediated inhibition of complex I may produce negative outcomes. For instance, a study performed in lipopolysaccharide (LPS)-induced rat model of PD showed that, despite some beneficial effects induced by metformin after the intranigral injection of LPS, such as the reduction of neuroinflammation, the drug not only did fail to rescue the dopaminergic neuronal loss induced by LPS but exacerbated it. The authors speculated that this result may be caused by the inhibition of complex I activity, affecting not only mitochondrial functionality but also cell homeostasis [17]. Along this line, metformin was shown to aggravate the dopaminergic neuronal loss in a PD mouse model based on the injection of 1-methyl-4-phenyl-1,2,3,6-tetrahydropyridine (MPTP), which inhibits mitochondrial complex I. In this case, the additive action of MPTP and metformin at the level of this complex and the consequent reduction of ATP may explain the detrimental effect of the drug [54]. Intriguingly, several PD models induced by the treatment with rotenone, which is another inhibitor of the complex I, showed opposite results. In fact, after rotenone injection in mice, metformin has been recently shown to reduce dopaminergic neuronal loss and rescue some PD behavioral phenotypes in mice [55]. Noteworthy, in a *C. elegans* model, it was observed that also mitochondrial hyperactivity may result in PD phenotypes, demonstrating that different alterations of mitochondrial homeostasis may be associated with PD pathology [19]. In this case, it is more intuitive to assume that the inhibitory effect mediated by metformin may be beneficial. Accordingly, metformin has been proven to rescue not only motor deficits but also the loss of dopaminergic neurons in *C. elegans* characterized by mitochondrial hyperactivation [19].

Besides the effects on the complex I activity, metformin has been also demonstrated to be important in maintaining mitochondrial quality control, improving mitochondrial fission, promoting respiration, and reducing oxidative stress [14,22]. For example, a recent work evaluated the effect of metformin in SH-SY5Y cells characterized by mitochondrial defects induced by the treatment with lead. In this model, the antidiabetic drug has been shown to reduce mitochondrial fragmentation and ameliorate mitochondrial morphology. Moreover, in the same cellular model, metformin has been described to reduce the mitochondrial-derived ROS levels induced by the treatment with rotenone. This antioxidant property of metformin has been associated with the activation of the nuclear factor erythroid 2-related factor 2 (Nrf2), which is a transcription factor responsible for the transcriptional regulation of genes involved in the response against oxidative stress. Moreover, the metformin-mediated Nrf2 modulation has been shown to be determined by the activation of Akt, an upstream activator of Nrf2 [16]. Interestingly, also AMPK activity is known to influence the Nrf2 pathway [56,57], suggesting that metformin may induce the antioxidant response through Nrf2. However, it is worthwhile to report that Akt and AMPK have also been reported to exhibit antagonistic activity in the ROS regulation, modulating different molecular pathways [58]. For this reason, it is crucial to understand whether metformin activates preferentially AMPK rather than Akt in particular conditions or at certain drug concentrations.

Hence, the ability of metformin to influence mitochondrial functions may be therapeutically relevant in the context of PD, in which mitochondrial defects and excessive oxidative stress are known to be associated with the pathology. Nevertheless, the contradictory results presented here demonstrate that further characterization of the role of metformin at mitochondria needs to be further elucidated.

### 2.4. Anti-Inflammatory Action

Accumulating evidence highlights the important role of non-neuronal cells in the onset and progression of neurodegenerative disorders, and glial cells’ activity has been widely studied in the PD context. These cells can regulate brain homeostasis and are essential for neuronal cell survival, modulating crucial processes, such as synaptic formation and maturation and response to stressful conditions [59,60]. Accordingly, several studies linked astrocyte dyshomeostasis to PD [61]. In particular, astrocytic activity has been evaluated in the case of neuroinflammation, one of the phenotypic traits associated with PD [62,63]. A study published in 2020 analyzed the effects of metformin on reactive astrocytes in a mouse 6-OHDA-based PD model, where the antidiabetic drug ameliorated astrocyte activation promoted by 6-OHDA and determined the downregulation of genes involved in astrocytes’ reactivity. These effects may be mediated by the induction of important cellular pathways, such as Akt, GSK3b, CREB, and BDNF-mediated pathways [26].

The microglial activity in the inflammation processes has been evaluated too, both in vitro and in rats and mice PD models. Interestingly, it was shown that metformin reduced microglial activation in cells and rats after LPS treatment. More specifically, metformin promoted the decrease of the number of activated glial cells, reduced the expression of pro-inflammatory mediators, and lowered the activation of the inflammasome as well as the accumulation of ROS in microglia [17]. The same effects have been observed in a mouse model following the treatment with MPTP [54].

Taken together, these results seem to underly the beneficial effect of metformin in reducing neuroinflammation, highlighting the importance of increasing the efforts in this research field to understand more in detail how the drug exerts the anti-inflammatory activity. Noteworthy, also the effects on microglial cells might be related to the activation of AMPK, which has been linked to the anti-inflammatory pathway, suggesting that the activation of the AMPK pathway may account for many of the effects ascribed to metformin [17,54,64].

## 3. Metformin as a Disease Modifier for Parkinson Disease: How Effective Is It?

### 3.1. Evidence from Epidemiological Studies on T2DM Patients

Based on the molecular mechanisms described so far, metformin appears of great interest as a translational approach for PD. Several studies performed on both in vitro and in vivo models of neurodegenerative diseases promisingly suggested that the metformin-mediated AMPK activation may reduce the level of neuronal loss and alleviate several phenotypes associated with these disorders. In light of these considerations, some epidemiological studies investigated in cohorts of T2DM patients the correlation between metformin therapy and the risk of developing PD (Table 3).

The first study, conducted in a large cohort of T2DM patients in Taiwan, found a 2.2-fold increased risk to develop PD in T2DM patients [65]. Interestingly, the treatment with sulfonylurea, another anti-hyperglycemic agent, significantly increased the risk of PD (HR 1.57, 95% CI 1.15–2.13), but it was alleviated for those patients who received a co-therapy with metformin (HR 0.78, 95% CI 0.61–1.01). However, the administration of metformin alone did not prove to prevent the development of PD in diabetic patients (HR 0.95, 95% CI 0.53–1.71) [65]. Later on, other clinical studies in Australia (2013), Norway (2017), and South Korea (2020) investigated the correlation between metformin therapy and PD, revealing a positive correlation with dementia, cognitive decline, and Parkinson syndrome [66,67,69]. Conversely, a longitudinal study in the USA in 2019 analyzed a 5-year follow-up in more than 5500 veterans with T2DM (around 60 years old), pointing out that a metformin therapy for more than 4 years significantly decreased the risk of developing both PD and AD (HR 0.19, 95% CI 0.12–0.31) [68]. Despite this promising avenue, a recent systematic review and meta-analysis by Qin and coworkers indicated an overall lack of correlation between metformin therapy and PD development (HR 1.23, 95% CI 0.98–1.78) [70]. More importantly, the sole exclusion of the study in the USA cohort resulted in a significant increase in PD risk (HR 1.50, 95% CI 1.11–2.02) [70]. Here, it must be acknowledged that these conflicting results may derive from a high degree of heterogeneity among clinical studies, in terms of population, treatment regime, follow-up lengths, and adjusted factors. In these studies, the average age of the enrolled subjects was 60–65 years, which corresponds to the mean age of onset of PD, although the incidence of PD increases with age (1–2% over 65 years old and more than 5% over 80 years old). However, the follow-up usually took place for an additional 6 years on average, possibly underestimating the correlation between metformin consumption and PD.

This notwithstanding, these clinical studies appear less promising than the large body of evidence of metformin neuroprotective effect in PD pre-clinical models. On these premises, a few aspects of metformin pharmacokinetics and pharmacodynamics need further consideration to evaluate the possible drawbacks of metformin prolonged therapy, namely, the bioavailability of the molecule in the brain and potential molecular side effects.

### 3.2. Metformin Bioavailability in the Brain

Metformin bioavailability has been determined to be around 40–60% [71] with a half-life of about 6.2 h in the plasma and an elimination half-life of 17.6 h [72]. The molecule does not encounter hepatic metabolism and it is eliminated mainly through the urinary tract in its unmodified form [42]. One of the still-unsolved aspects regards the concentration and accumulation of the drug in the brain after administration and how metformin reaches the brain tissue. In this context, several works demonstrated that metformin can penetrate the blood–brain barrier (BBB) in mammals, as indicated by the presence of the molecule in the cerebrospinal fluid (CSF) of rats after oral administration [73,74]. Along this line, another important factor to be assessed is the brain-to-plasma ratio of metformin, as this parameter indicates the ability of the molecule to pass through the BBB. This ratio has been evaluated independently in rats by two research groups that obtained different results. In the first publication, a rat model of inflammation induced by LPS showed that the brain-to-plasma ratio reached the maximum value 6 h after single oral administration. At this time point, the concentration of metformin in the blood plasma was comparable to the concentration in the brain, suggesting a high permeability of the drug across the BBB [73]. The same study also showed that the concentration of metformin was not the same in different brain regions. Moreover, acute and chronic administrations induced a different pattern of distribution with the highest concentration of the molecule in the cerebellum and the pituitary gland, respectively [73]. Conversely, the other study found in the CSF of rats only 4% of the plasma metformin concentration, suggesting a low ability to cross the BBB [74]. Even though these contradictory results may be explained by different methods used for the analysis or by variations in the method of administration, these data prompt the need to unequivocally estimate the percentage of metformin that penetrates the diverse brain districts.

Another critical factor that must be evaluated is the mechanism that governs the uptake of metformin within neuronal cells. Since metformin is positively charged it is improbable that it can easily pass the plasma membrane through simple diffusion [75]. Therefore, it is most likely that some membrane transporter plays a crucial role in the absorption and distribution of metformin in cells. In this frame, several studies confirmed that metformin is a substrate of numerous organic cation transporters (OCTs), including OCT1, OCT2, OCT3, MATE1, MATE2, PMAT, and OCTN1 [76]. These transporters are responsible for the recognition and transport of a broad variety of molecules and drugs, which are positively charged at physiological pH [77]. The pattern of expression of these transporters may highly influence the rate of metformin absorption in different tissues. For this reason, the analysis of the distribution of OCTs in the brain, with a specific focus on the expression of these transporters at the BBB level, as well as in different brain regions and different cell types, may be crucial to characterize the impact of metformin on brain physiology. An interesting work published in 2020 analyzed the expression of several OCTs in the BBB of rats, mice, and humans both in vivo and in vitro, using primary cell cultures [78]. The presence of the transporters was analyzed through the analysis of the OCTs’ mRNA levels, and the authors performed also functional analysis evaluating the differences in permeability of known substrates through the membranes in the presence or absence of inhibitors of the transporters. This study revealed the lack or a negligible presence of OCT1, OCT2, OCT3, and MATE1 in the BBB of all organisms [78]. These results were quite unexpected considering the aforementioned ability of metformin to cross the BBB. This aspect may be partially explained by the fact that not all the OCTs responsible for metformin transport were evaluated in the study. Another possibility is that other unknown transporters in the BBB determine the passage of metformin. Further analyses are necessary to assess how metformin can reach the brain tissue. In contrast to the reported study, a previous work conducted in 2010 used confocal imaging and Western blot analysis to evaluate the presence of OCTs in the BBB. The authors found that both OCT1 and 2 are detectable in the BBB of rats, mice, and humans [79]. The discrepancies in the results of different scientific works are difficult to explain, but it is important to note that the different techniques used may have different levels of resolution. Moreover, the presence of the transporters detected in the latter study may also be determined by the signal contamination caused by other cells, such as neurons, in which the presence of OCTs seems to be more abundant. In fact, OCT2, OCT3, OCTN1, and, to a lesser extent, OCT1 expression has been demonstrated in human and rodent neurons and may account for the intake of metformin in neuronal cells [80,81].

To assess whether metformin can be therapeutically beneficial for neurodegenerative diseases it is also crucial to determine the concentration of the drug into the brain that is necessary to promote protective effects and to understand the appropriate way to administer the molecule to gain the region of interest in the brain as well as inside neurons and glial cells. In this frame, the group of Kalyanaraman developed mitochondrial-targeted analogs of metformin (mito-metformin) by attaching a positively charged lipophilic triphenylphosphonium group to the molecule, thus increasing metformin bioavailability in the subcellular compartment of about 1000 times fold [82]. Despite being conceived to increase the anti-tumor potential of metformin [83], the administration of mito-metformin to the MitoPark transgenic mouse model of PD (knockout mouse for the mitochondrial transcription factor A in midbrain dopaminergic neurons) was able to promote mitophagy, restore striatal dopamine levels, and rescue both the motor and behavioral phenotype [84]. Moreover, the investigators tested the efficacy of mito-metformin in a cellular model of rotenone-induced mitochondrial dysfunction, revealing that the delivery of the drug by functionalized polyanhydride nanoparticles (NPs) provided a significant amelioration of cell viability at nanomolar concentrations of mito-metformin [85].

### 3.3. Side Effects Associated with Prolonged Metformin Consumption

Metformin is generally well tolerated with minimal side effects for the majority of medication users. The most frequent adverse effects are related to hypoglycemia and irritation of the gastrointestinal tract for which the subjects can experience bloating, flatus, diarrhea, nausea, and constipation. About 50% of T2DM patients under metformin therapy have been diagnosed with plasma acidosis [72]. This condition is defined by plasma pH < 7.3 and a lactate concentration >5.0 nmol/L, and, in the case of metformin, this is likely to derive from the alteration of glucose metabolism in hepatocytes, resulting in increased conversion of pyruvate to lactate, which is further released in the bloodstream. Although only a small percentage of cases of plasma acidosis is life-threatening due to complete kidney failure, reduced renal functions have been associated with prolonged consumption of metformin at high dosage (>2 g/day, when the average dose for T2DM patients is 500–1700 mg/day) for patients older than 65 years [72].

More importantly, a severe vitamin B12 (VitB12) deficiency has been found in metformin users [72]. VitB12 or cobalamin is an important cofactor for enzymes involved in the DNA synthesis and fatty acids’ and amino acids’ metabolism, as well as enzymes involved in neuroprotective functions, myelin synthesis, and blood cell maturation in the bone marrow. A decreased intestinal uptake of VitB12 results in peripheral neuropathy, axonal demyelination, and hematological abnormalities [72]. In a recent retrospective cohort study on patients with more than 1 year of metformin consumption, around 3.3% of the subjects displayed a significant VitB12 deficiency, with the highest correlation in people over 80 years old (63%) [86]. Although the precise mechanism of metformin inhibition of VitB12 uptake has not been fully elucidated, it might depend on an interference with the calcium-dependent binding of the gastric intrinsic factor-VitB12 complex with the cubilin receptors on enterocytes at the level of the ileum, a direct interaction between metformin and the cubilin receptor, which blocks VitB12 uptake, or a dysregulated intestinal microbiota outgrowth, which potentially affects cubilin receptor accessibility by VitB12 [87].

Interestingly, a case-control study carried out in Australia between 1998–2008 positively correlated the VitB12 deficiency concomitant to metformin consumption with increased risk of cognitive impairment and AD in T2DM patients over 65 years old (HR 1.71, 95% CI 1.12–2.60) [69]. The correction of the VitB12 levels in the plasma by calcium supplementation to promote VitB12 uptake was able to preserve the cognitive functions [69]. In addition, a recent study on idiopathic PD patients in Korea correlated VitB12 deficiency (<133 pg/mL) not only with cognitive impairment but also with the decreased motor performance of the individuals, while VitB12 supplementation was able to provide significant improvement of the motor symptoms [88]. On the same line, gastric cancer patients subjected to total gastrectomy (thus preventing the physiological VitB12 uptake) displayed a higher risk of developing PD (HR 1.55, 95% CI 1.03–2.32), whereas introducing VitB12 supplement significantly reduced the risk of PD of 60–70% (HR 0.36, 95% CI 0.17–0.76) [89].

Noteworthy, another recent cross-sectional analysis on PD patients from DATATOP [90] assessed the levels of VitB12 deficiency, indicating that 13% of patients presented VitB12 plasma concentration below the minimum threshold of 250 pg/mL [91]. This value decreased with aging between −17 and −47 pg/mL/year (which is more than the average VitB12 decrease of −5 pg/mL/year in the healthy elderly population) and was correlated with a progressive decrease in patients’ motor performance (gait impairment). Moreover, at the time of diagnosis, PD patients presented the lowest VitB12 concentration as compared to other neurodegenerative disorders, i.e., AD, MSA, progressive supranuclear palsy, frontotemporal dementia, DLB, and mild cognitive impairment [91]. The authors discussed the possible causes of the decreased uptake of VitB12 at the gastrointestinal level in PD patients, suggesting, among others, the infection by *H. pylori*, a delayed gastric emptying, constipation, and bacterial outgrowth [91]. This is particularly relevant in light of the gut–brain axis involvement in PD, with the suggested possibility that the primary site of PD pathology could originate in the enteric system due to microbial dysbiosis and α-syn enteric aggregation and then propagate to the central nervous system through the vagal nerve [92]. Coincidently, it has been demonstrated that VitB12 negatively regulates α-syn fibrillation by direct binding to the protein and it reduces α-syn-induced cytotoxicity [93].

On these premises, the VitB12 deficiency and the gastro-intestinal alterations induced by metformin consumption should be taken into consideration during prolonged therapies with metformin. This is especially relevant when high dosages are required to ensure a proper bioavailability of the drug in the brain because they might exacerbate VitB12 depletion in elderly individuals who already have an increased risk of VitB12 deficiency at the age of PD clinical onset [87]. In addition, despite the putative role of metformin in counteracting α-syn spreading, it might have an indirect pro-aggregating action on the enteric α-syn, thus accelerating the early events of PD pathology.

### 3.4. Metformin Treatments in Experimental Models versus Human Subjects: Looking for a Key of Interpretation

An important aspect that still needs to be elucidated is whether metformin consumption in healthy individuals might be beneficial in targeting and delaying aging, as well as age-related disorders [94]. Indeed, potential metformin-dependent side effects displayed by T2DM patients might be absent in a diabetic-free background. However, at present, no clinical data are available aiming at assessing a decreased risk of PD other than for T2DM patients under the metformin regime.

Anyway, a few studies of in vivo experimental models investigated the role of metformin per sè in brain bioenergetic and behavioral response. Specifically, when 10-week-old mice were injected with 200 mg/kg/day of metformin, the activation of AMPK and the levels of GDNF and BDNF were significantly higher both in SNpc and striatum, resulting in TH upregulation and improved dopaminergic neuron health [95]. In another study, 4-month-old mice subjected to chronic administration of 180 mg/kg/day of metformin (which, according to the authors, should correspond to about 900 mg/day intake for humans) displayed an improved motor performance but cognitive impairment [96].

It is worth mentioning that, in both studies, the effects of metformin were tested in relatively young mice and in quantities that correspond, at least in theory, to the minimum clinical dosage. It has been estimated that in humans the plasma concentration of metformin ranges between 10 µM and 40 µM when assuming 1 g of drug per day [71]. However, the equivalence between the dosage in humans and the concentrations used in experimental models is still under debate, even though this would be extremely relevant to accurately correlate the molecular readouts detected in research models with the effects observed in humans. According to Lamoia and colleagues, the inhibition of mitochondrial complex I and AMPK activation detected in cellular models treated with millimolar concentrations of metformin correspond to supra-pharmacological concentrations, whereas the redox balance is stimulated at micromolar concentrations, defined as clinically relevant doses [71].

Another key issue is the inconsistency of dosage among the experimental studies present in the literature, together with an extremely high variability of effects according to the model system and the readouts considered. This is exacerbated when the metformin neuroprotective role is tested in both in vitro and in vivo PD models, where the neurodegenerative phenotype is induced either genetically (i.e., α-syn overexpression) or pharmacologically by LPS or neurotoxins such as 6-OHDA, rotenone, and MPTP. Indeed, since metformin has multiple direct and indirect targets (Figure 1), its mechanisms of action may significantly vary according to the animal model, the concentration, the administration route, and PD stimulus, thus challenging data interpretation and translation to humans. An example is represented by the inhibitory activity on mitochondrial complex I, which remains to be unresolved whether it is beneficial or not, in particular in the PD context. The controversial results present in the literature may depend on the amount and the duration of metformin treatment, as well as the PD model, as exemplified by the conundrum on the aggravating effect in the MPTP model versus the neuroprotective effect in the rotenone one [54,55]. Moreover, the different outcomes may be also due to different levels of complex I inhibition in different experimental models. A mild complex I inhibition might be advantageous as a reduced ATP/ADP ratio activates the AMPK signaling pathway, thus promoting autophagy-mediated degradation of misfolded proteins and dysfunctional organelles. Moreover, a partial complex inhibition could maintain under control the redox state of mitochondrial along with the dopaminergic neuronal projections that present higher rates of oxidative phosphorylation [97]. Conversely, an excessive inhibitory effect on complex I, comparable to the enzyme ablation, has been demonstrated to be sufficient in triggering a PD phenotype [53]. In addition, several contradictory results highlighted in some reports further complicate the analysis and the precise understanding of metformin activity at the mitochondria. For example, a recent paper demonstrated that low metformin concentrations may stimulate mitochondrial respiration and complex I activity in mice rather than inhibiting it [14]. Therefore, it is necessary to understand the precise metformin dosage necessary to alter the AMP/ATP ratio and activate AMPK, without the induction of detrimental effects.

## 4. Conclusions

Several works recently suggested that metformin may be used as a promising therapeutic strategy to counteract the progression of neurodegenerative disorders, including PD. However, despite intensive research, the precise mechanisms by which metformin exerts its activity are not completely understood. Therefore, to determine the potential therapeutic use of metformin as a disease modifier for PD, it is crucial to evaluate its main pharmacological properties, such as the bioavailability in the brain, the molecular pathways it affects, and the concentrations necessary to observe the beneficial effects. Unfortunately, the data available in the literature are often controversial and vary significantly among experimental models, prompting the necessity to outline a consensus in pre-clinical data interpretation.

At the same time, the prolonged consumption of metformin at a relatively high dosage might induce serious side effects that could worsen the risk of developing PD over time. In this frame, the epidemiological studies that investigated the association between metformin therapy and PD only assessed the effect of the drug in T2DM patients [65,66,67,68,69]. Additionally, to our knowledge, phase 1 clinical trials of metformin did not provide an appropriate follow-up on the enrolled subjects aiming at verifying a decreased incidence of PD with aging. Thus, it remains to be evaluated by ad hoc clinical studies whether metformin administration to non-diabetic subjects exerts a neuroprotective activity towards PD and other neurodegenerative disorders. In this case, the ability of metformin to effectively act as a disease modifier might depend on an early intervention in the prodromal phases of PD, when the neurodegenerative process is not irreversibly advanced. Hence, the development of new criteria for patients’ stratification strategies should be a primary goal to identify those individuals who could benefit most from metformin therapy in the long run [42].

## Figures and Tables

**Table 1:** Molecular read-outs of metformin administration to non-PD and PD cellular models.

**Cellular Model**	**Concentration**	**Duration of Treatment**	**Read-Out**	**Ref.**
Human neuronal stem cell	1 mM	48 h	− increased cell viability − increased mitochondrial functions − AMPK activation	[10]
Rat hepatocytes	0.05–0.1 mM	24–60 h	− inhibition of mitochondrial respiration − inhibition of complex I in isolated mitochondria	[11]
Rat hepatocytes	From 0.02 to 2 mM	1–7–39 h	− AMPK activation	[12]
Mitochondria isolated from frontal mouse brain	1 mM	15 min	− inhibition of complex I − reduced mitochondrial respiration	[13]
Mouse primary hepatocytes	0.5–1 mM	22 h	− reduced adenine nucleotides − reduced mitochondrial respiration	[14]
Mouse primary hepatocytes	0.075 mM	22 h	− increased mitochondrial respiration − increased mitochondrial fission − increased AMPK activity	[14]
**In vitro** **PD cellular models**				
SH-SY5Y overexpressing α-syn	0.5–1.0–2.5 mM	16–24 h	− PP2A activation and reduced α-syn pSer129 − AMPK activation and mTOR inhibition	[15]
SH-SY5Y neuroblastoma cells treated with rotenone	From 0.01 to 10 mM	2–3–6 h	− reduced cell death − reduced caspase 3/7 activity − AMPK activation − reduced mitochondrial ROS production − prevented antioxidant depletion and mitochondrial dysfunction − induced Nrf2 pathway via Akt	[16]
BV2 cells treated with LPS or IL-4	1 mM	3–12–24 h	− reduction of microglial activation − reduction of ROS − reduction of NADPH oxidase activity	[17]

**Table 2:** Molecular read-outs of metformin administration to non-PD and PD animal models.

Animal Model	Dosage	Duration	Administration Route	Read-Out	Ref.
*C. elegans*	50 mM		Oral	− increased lifespan − improved fitness − inhibition of TORC1 pathway and activation of the lysosomal pathway − activation of AMPK	[18]
*C. elegans* bcat-1 knock-down	0.05 mM	4 days	Oral	− complex I inhibition − rescue neuronal viability	[19]
*D. melanogaster*	From 1 to 100 mM	7 days	Oral	− AMPK activation − reduction of fat stores − disruption of intestinal fluid homeostasis	[20]
*D. melanogaster*	5 mM	7 days	Oral	− inhibition of age-related centrosome amplification in midgut stem cells − inhibition of Akt/TOR pathway	[21]
*M. musculus*	0.1–1% *w*/*w*		Oral	− increased lifespan and health span − mimicking of calorie-restriction transcriptome − Activation of AMPK − activation antioxidant response	[22]
*M. musculus*	200 mg/Kg		Intraperitoneal injection	− increased Ach levels − decreased choline levels	[13]
*M. musculus*	1–10 mM		Hypothalamus infusion	− increased Ach levels − decreased choline levels	[13]
*M. musculus*	From 6.25 to 50 mg/kg	Once a day for 12 weeks	Oral	− activation of complex I (50 mg/kg) − activation of AMPK	[14]
*R. norvegicus*	50–150 mg/ml	Once a day for 5 days	Oral intubation	− reduction of ATP/ADP ratio in the liver − change of glycolytic metabolite	[11]
**In vivo** **PD animal models**					
*C. elegans* treated with 50 mM 6-OHDA	5–10 mM	72 h	Oral	− increased lifespan − decreased degeneration of dopaminergic neurons − reduced α-syn aggregation − upregulation of dopamine synthetic gene cat-3 and antioxidant gene sod-3	[23]
*M. musculus* injected with MPTP (30 mg/kg/day) for 7 days	200 mg/kg/day	7 days (following MPTP)	Intraperitoneal injection	− recovery from motor dysfunction − increased TH expression in the striatum and restored dopamine levels − decreased caspase-3 and apoptosis inhibition − reduced astroglia activation − AMPK activation and mTOR inhibition − PP2A activation and reduced α-syn pSer129 − upregulation of neurotrophic factors (BDNF, GDNF) and activation of downstream signaling pathways (Akt, Erk1/2)	[24]
*M. musculus* injected with rotenone (2.5 mg/kg/day) for 10 days	300 mg/kg/day	10 days (co-admin.)	Intraperitoneal injection	− rescued dopaminergic neuron loss in SNpc − decreased caspase-3-mediated apoptosis − reduced α-syn accumulation − decreased levels of lipid peroxidation products (4-HNE, MDA) − no difference in motor behaviors	[25]
*M. musculus* treated with 15 μg 6-OHDA	100–200 mg/kg	Once a day for 4 weeks	Oral	− rescue of motor deficits − induced AMPK, AKT, BDNF, GSK3b, CREB pathway − reduced astrocyte activation	[26]
*R. norvegicus* injected with 2 μg of LPS	150 mg/Kg	Twice a day for 7 days	Oral	− reduction of the number of activated microglial cells − reduction of inflammatory mediators and microglial pro-inflammatory phenotypes − decreased activation of the inflammasome	[17]

**Table 3:** List of epidemiological studies on T2DM patients treated with metformin evaluating the association with the risk of PD.

Location	Study Period	Sample Size	Medication Users	Mean Age (Years)	HR [95% C.I.]	Follow-Up (Years)	Reference
Taiwan	1996–2007	11,730	1879	64.3 ± 9.6	0.95 [0.53–1.71]	11 or until PD onset	[65]
Norway	2004–2014	102,745	94,349	63.4 ± 11.1	1.39 [1.06–1.82]	6.95	[66]
South Korea	2009–2010	1,308,089	644,921	60.8 ± 10.0	1.22 [1.10–1.36]	6.3	[67]
USA	2004–2010	5530	2774	63.2 ± 10.9	0.19 [0.12–0.31]	5.2	[68]

## Data Availability

Not applicable.
